# Supplementary figures and images for: Loss of FIC-1-mediated AMPylation activates the UPRER and upregulates cytosolic HSP70 chaperones to suppress polyglutamine toxicity
Source: PLoS Genet. 2025 Jun 13;21(6):e1011723. doi: 10.1371/journal.pgen.1011723 (PMC12193957; doi:10.1371/journal.pgen.1011723)

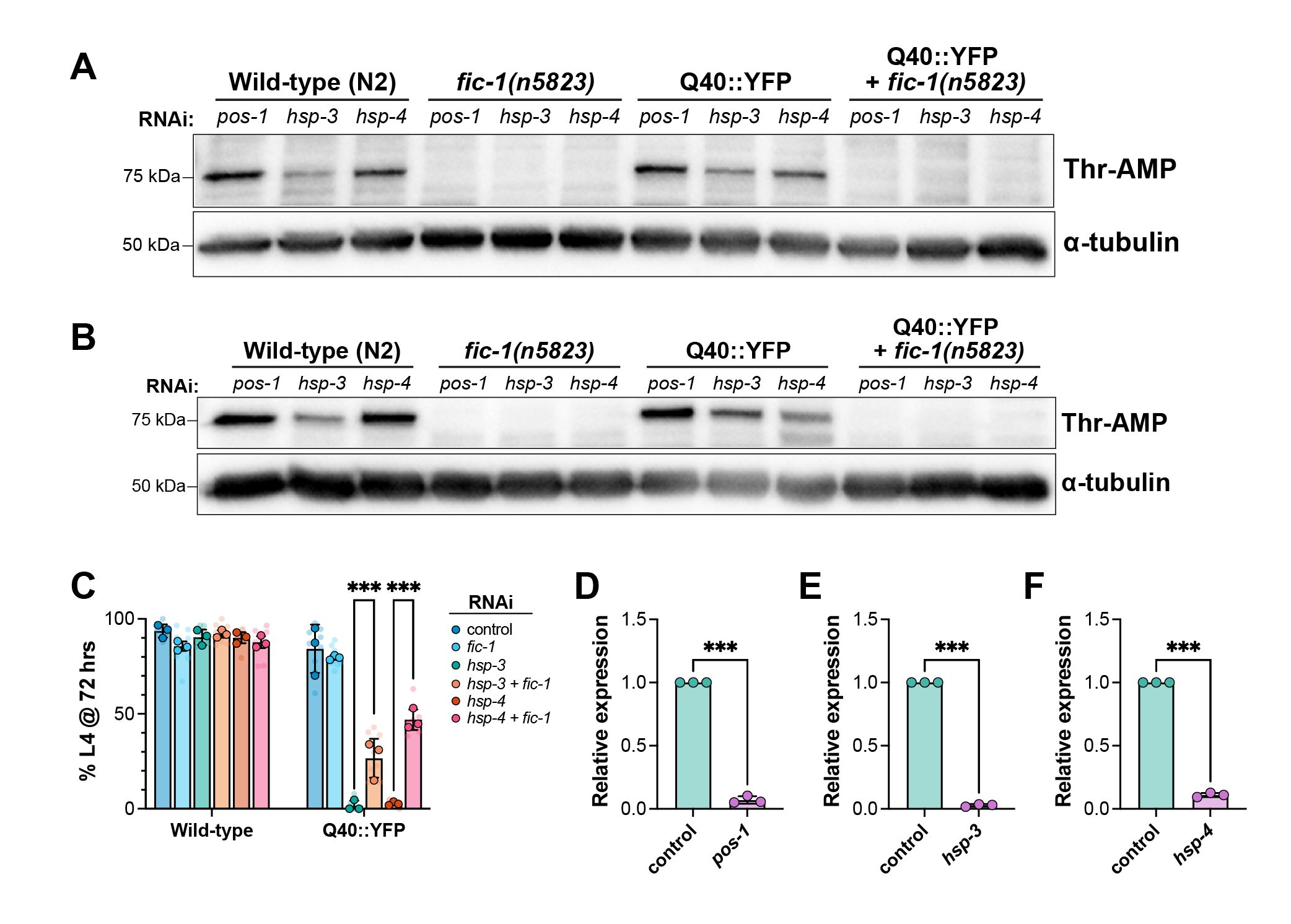

Supplement: S1 Fig — These blots represent additional biological replicates of the data depicted in Fig 1A and are included in the quantification of Thr-AMP signal shown in Fig 1B. (C) Development assay indicating the proportion of wild-type or Q40::YFP animals surviving to the L4 stage of development after 72 hours at 20ºC when fed the indicated RNAis (legend) from hatching. (D-F) Relative mRNA expression levels of the pos-1 (D), hsp-3 (E), and hsp-4 (F) when animals were fed RNAi against the indicated gene from hatching, analyzed by qPCR. Error bars for all plots represent SD. For C, a two-way ANOVA with Tukey’s post-hoc multiple comparisons tests was performed to determine statistical significance. For (D-F), statistical significance was calculated using an unpaired T-test. ***p < 0.001; ns = not significant. (TIF) [file pgen.1011723.s001.tif]

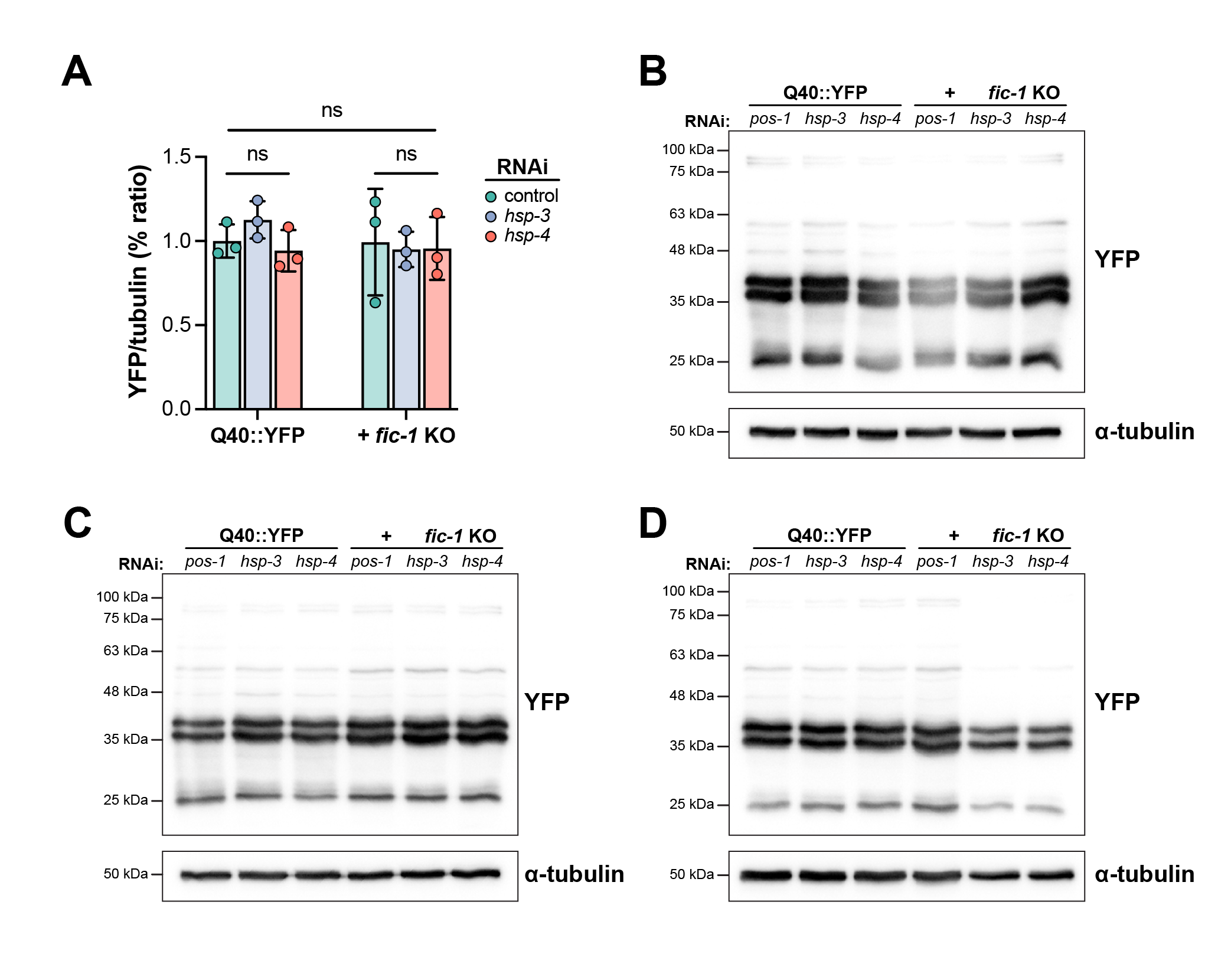

Supplement: S2 Fig — These blots represent 3 biological replicates. (D) Quantification of Q40::YFP expression levels expressed as a percent ratio normalized to α-tubulin. Errors bars represent SD. A two-way ANOVA with Tukey’s post-hoc multiple comparisons tests was performed to determine statistical significance. ns = not significant. (TIF) [file pgen.1011723.s002.tif]

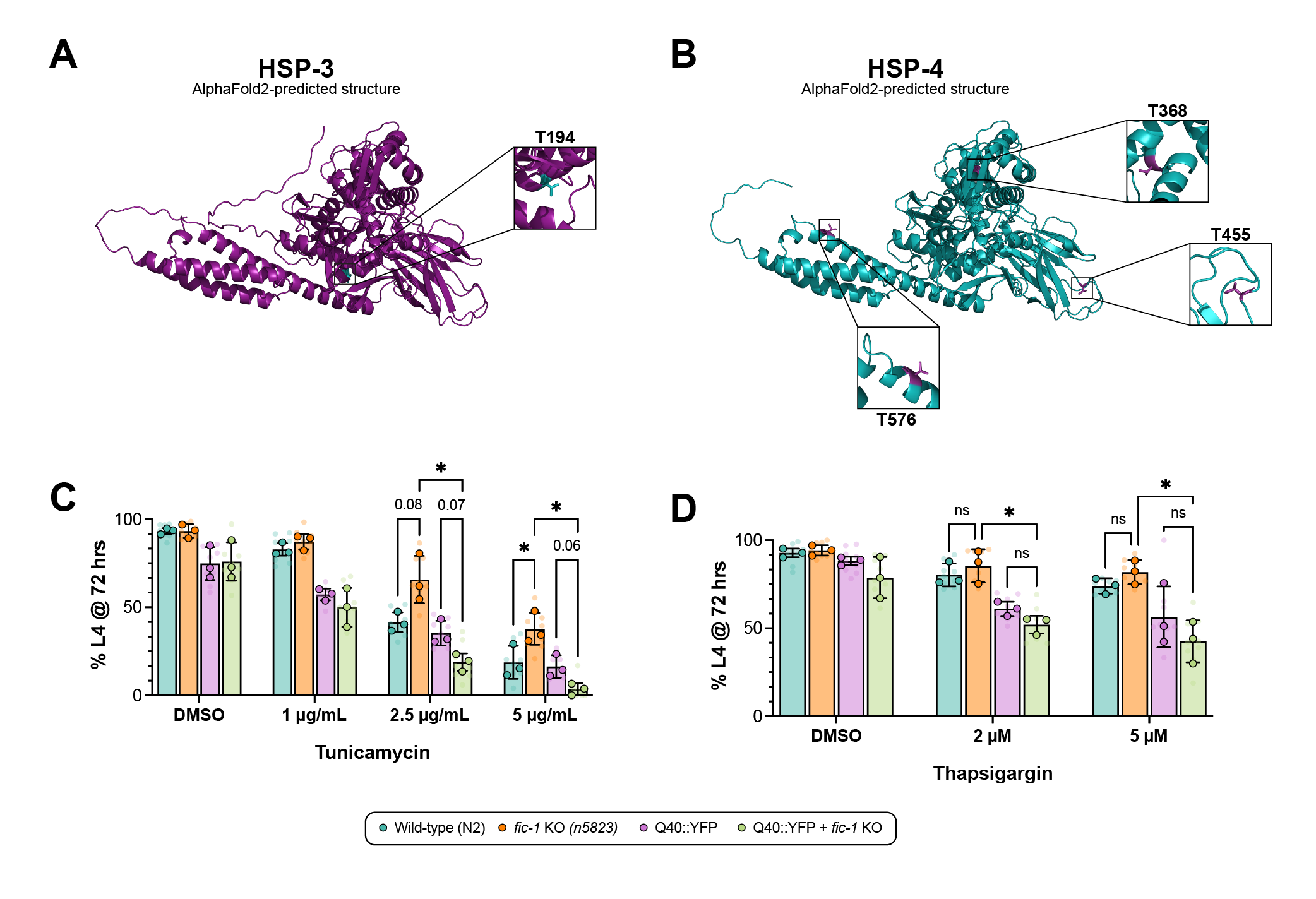

Supplement: S3 Fig — (C-D) Development assays of the indicated strains (see legend) depicting the percentage of animals reaching the L4 stage of larval development at 72 hours in the presence of the ER stressors (C) tunicamycin or (D) thapsigargin. X-axes denote the control (DMSO) and concentrations tested. For (C-D), translucent data points reflect technical replicates, while opaque data points depict the average of each biological replicate (n = 3). For (C-D), two-way ANOVAs with Tukey’s post-hoc multiple comparisons tests were performed to determine statistical significance. *p < 0.05; ns = not significant. (TIF) [file pgen.1011723.s003.tif]

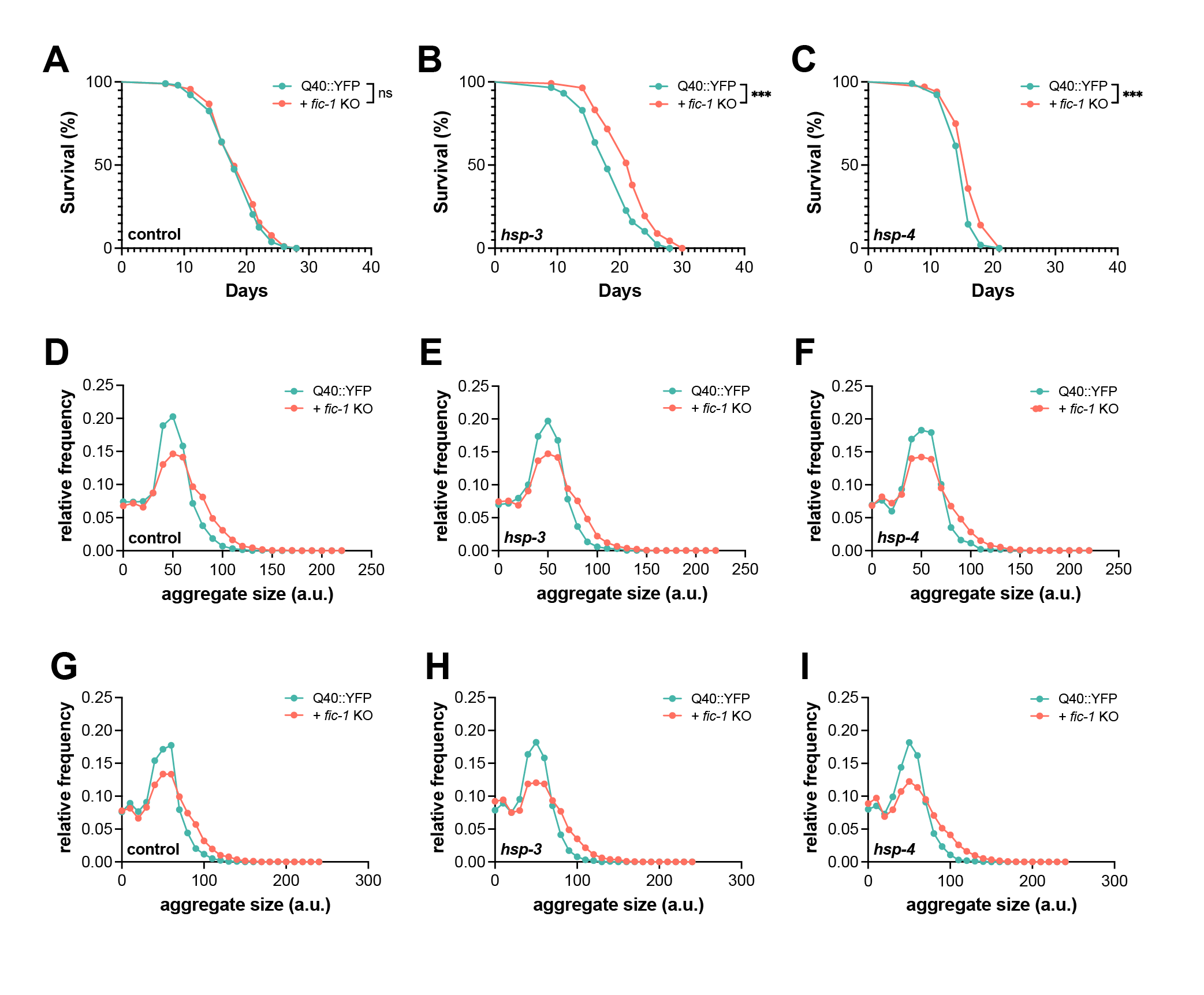

Supplement: S4 Fig — These graphs represent additional biological replicates of the lifespan curves shown in Fig 2. A Mantel-Cox test was used to determine statistical significance. (D-F) Frequency distribution profiles of polyQ puncta sizes in day 3 adult worms fed control (D), hsp-3 (E), or hsp-4 (F) RNAi. Bin size = 50 a.u. (G-I) Frequency distribution profiles of polyQ puncta sizes in day 5 adult worms fed control (G), hsp-3 (E), or hsp-4 (F) RNAi. Bin size = 100 a.u. ***p < 0.001; ns = not significant. (TIF) [file pgen.1011723.s004.tif]

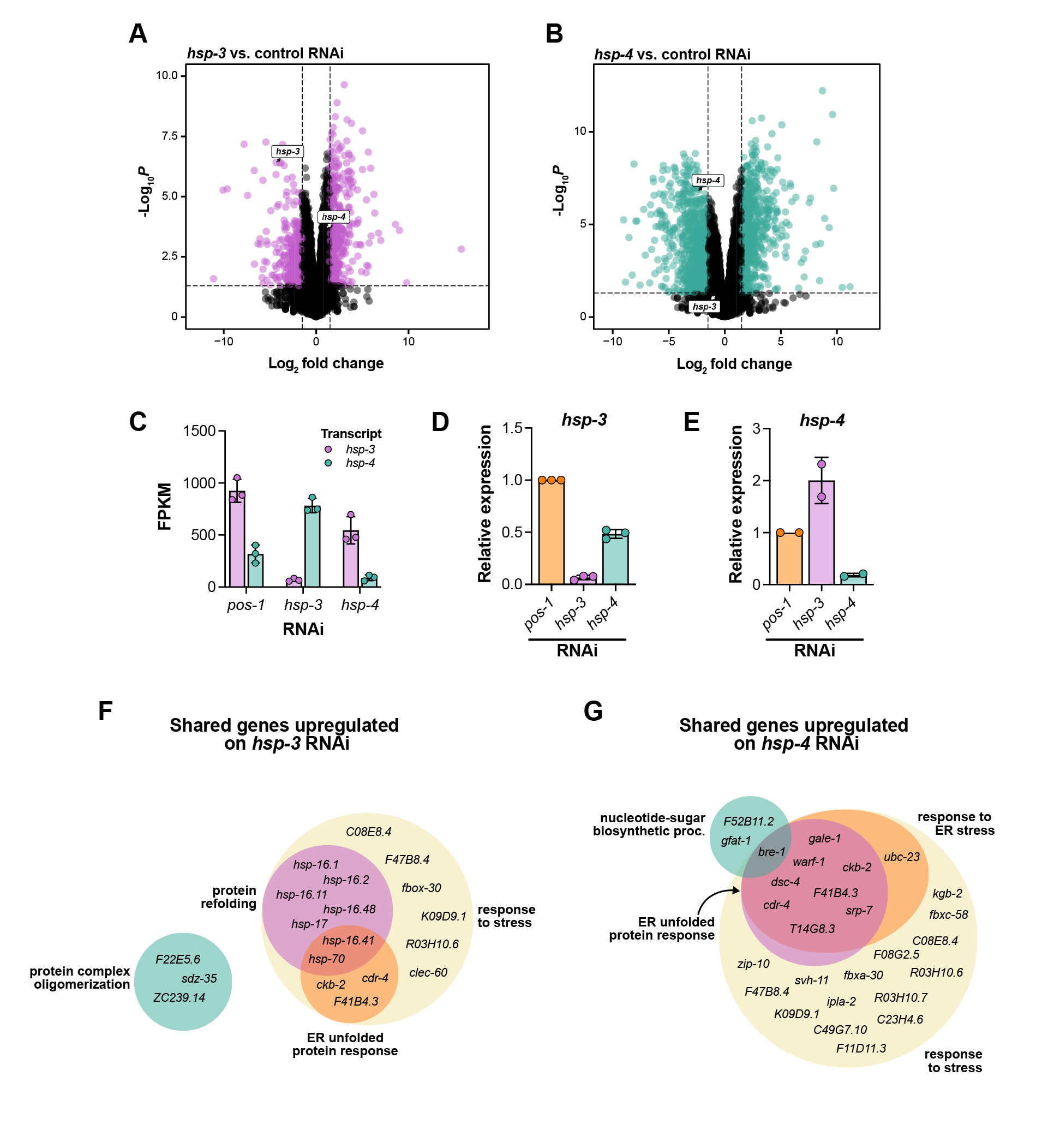

Supplement: S5 Fig — Cut-offs (dashed lines): log2FC > 1.5, p < 0.05. (C) Fragments per kilobase of transcript per million mapped reads (FPKM) values for hsp-3 and hsp-4 transcripts in Q40::YFP; fic-1 KO animals fed the indicated RNAis (X-axis). Each data point reflects one biological replicate (n = 3). (D-E) Relative hsp-3 (D) and hsp-4 (E) expression levels in animals fed the indicated RNAis (X-axis). (F-G) Visual depiction of commonly upregulated genes across all genotypes on (F) hsp-3 or (G) hsp-4 RNAi vs. control, grouped by gene ontology (GO) biological process terms. (TIF) [file pgen.1011723.s005.tif]

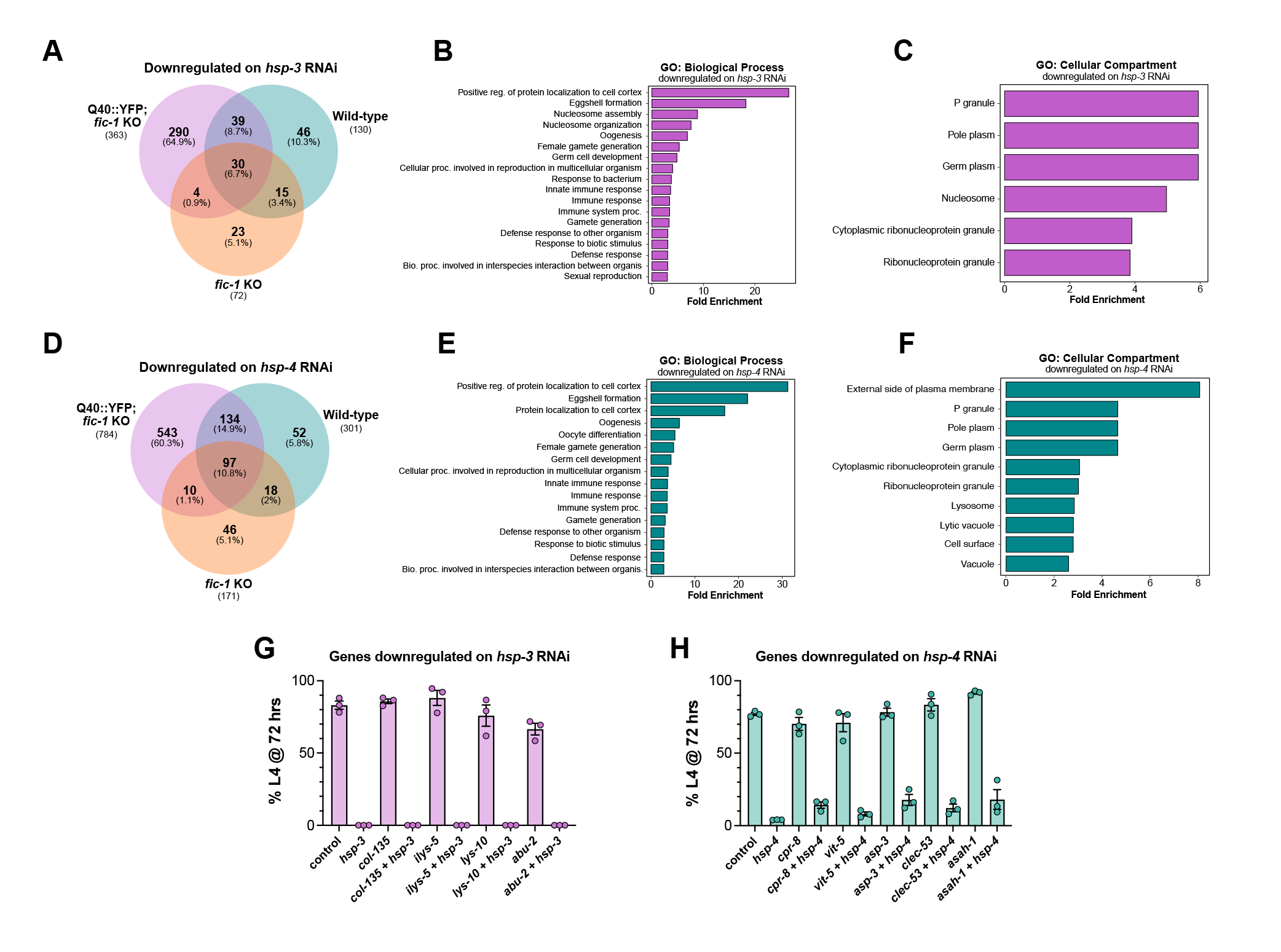

Supplement: S6 Fig — (B) Over-represented gene ontology (GO) biological process terms downregulated under hsp-3 knock-down conditions, ordered by fold enrichment. (C) Over-represented GO cellular compartment terms downregulated upon hsp-3 knock-down, ordered by fold enrichment. (D) Venn diagram of genes downregulated in response to hsp-4 knock-down, with 543 genes specific to Q40::YFP; fic-1 KO animals. (E) Downregulated GO biological process terms under hsp-4 knock-down conditions, ordered by fold enrichment. (F) Downregulated GO cellular compartment terms in response to hsp-4 knock-down, ordered by fold enrichment. (G-H) Limited screen of genes downregulated on hsp-3 (G) and hsp-4 (H) RNAi performed in Q40::YFP animals. X-axes indicate the RNAi conditions used. Each plot depicts the percentage of animals that have reached the L4 stage of larval development when assessed at 72 hours. For (G-H), each data point reflects one plate, or technical replicate. (TIF) [file pgen.1011723.s006.tif]

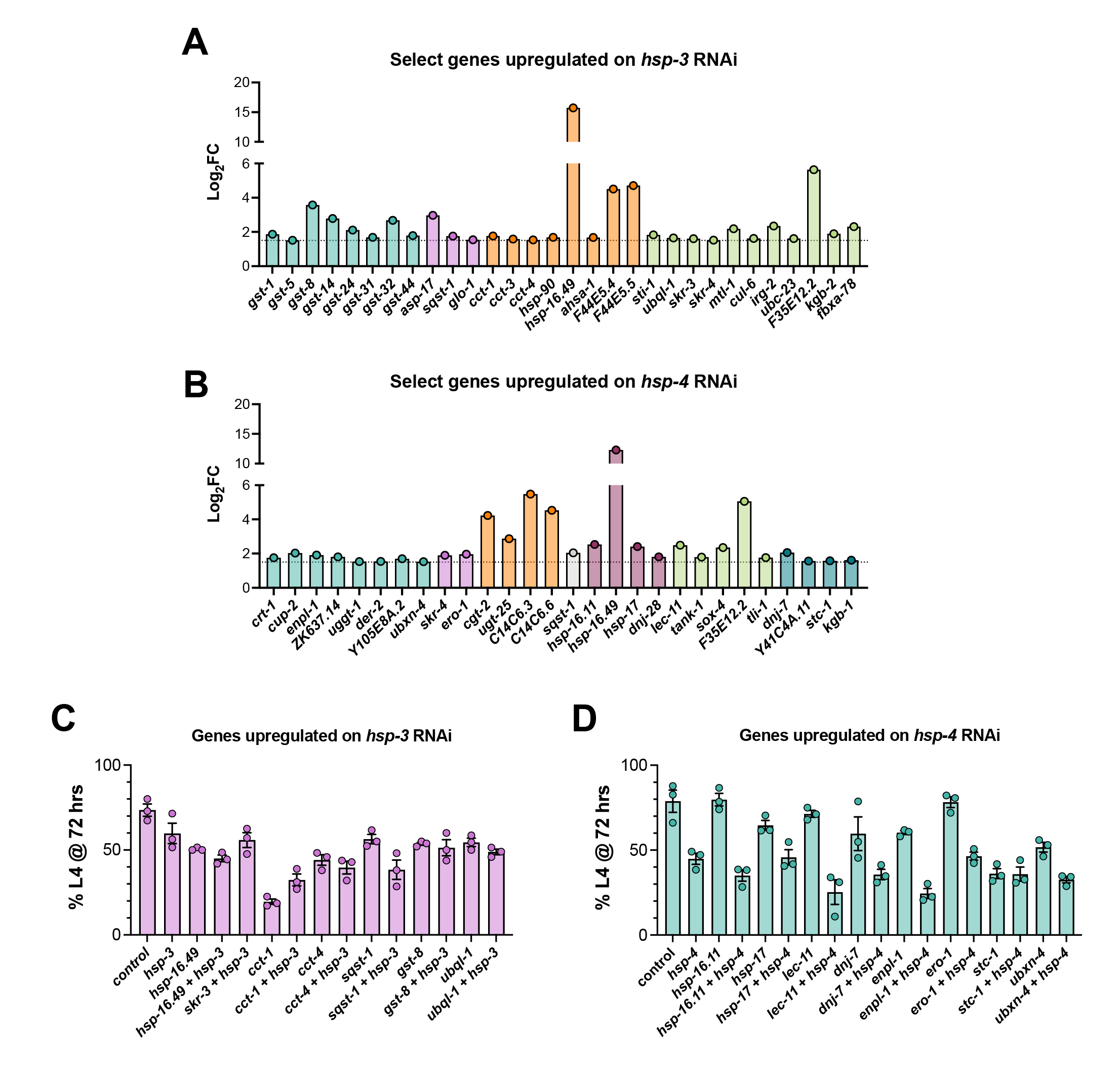

Supplement: S7 Fig — These plots correspond with the heat maps shown in main-text Fig 3I - 3J. Genes are colored according to functional grouping (from left to right, A: glutathione metabolism, lysosome, protein folding, stress response; B: ERAD pathway, ER stress and protein processing, glycotransferase activity, lysosome, protein folding, stress response, and UPR). (C-D) Limited screen of genes upregulated on hsp-3 (C) and hsp-4 (D) RNAi performed in Q40::YFP; fic-1 KO animals. X-axes depict RNAi conditions used. Each plot shows the percentage of animals that have reached the L4 stage of larval development when assessed at 72 hours. For (G-H), each data point reflects one plate, or technical replicate. (TIF) [file pgen.1011723.s007.tif]

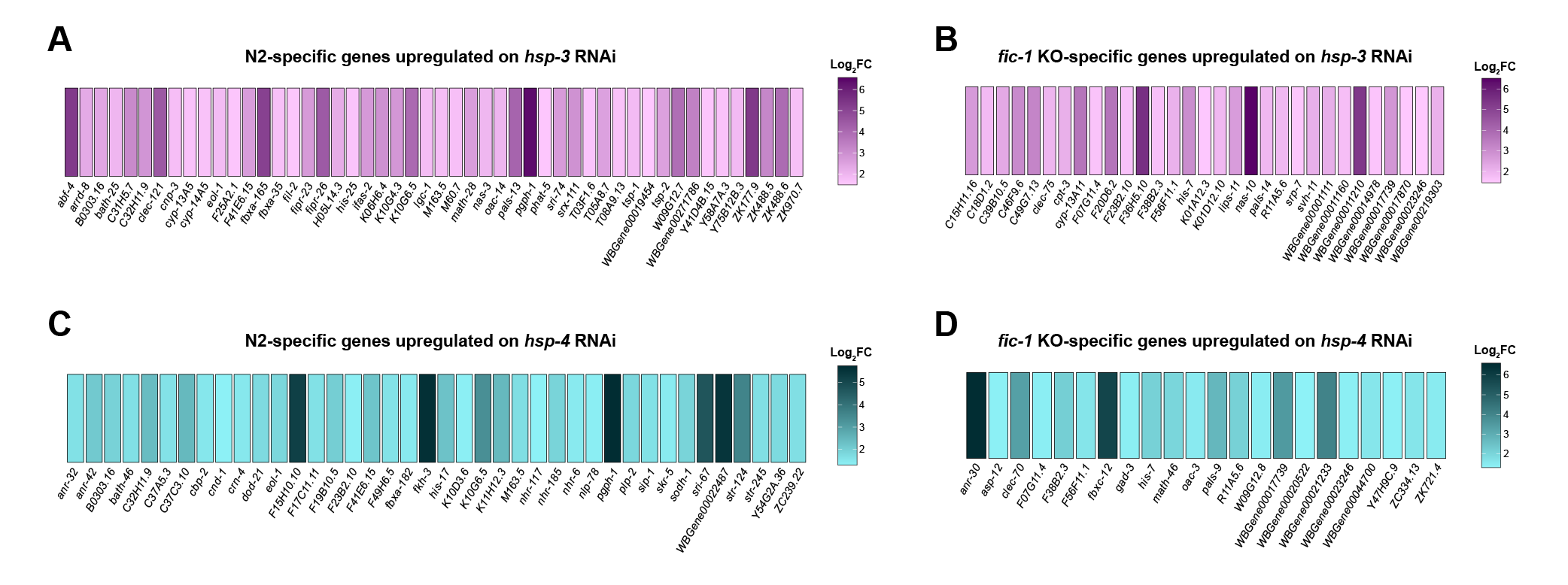

Supplement: S8 Fig — (C-D) Heat maps of genes specific to (C) wild-type (N2) or (D) fic-1 KO (n5823) animals on hsp-4 RNAi vs. control. For all graphs, genes are colored according to log2FC values. (TIF) [file pgen.1011723.s008.tif]

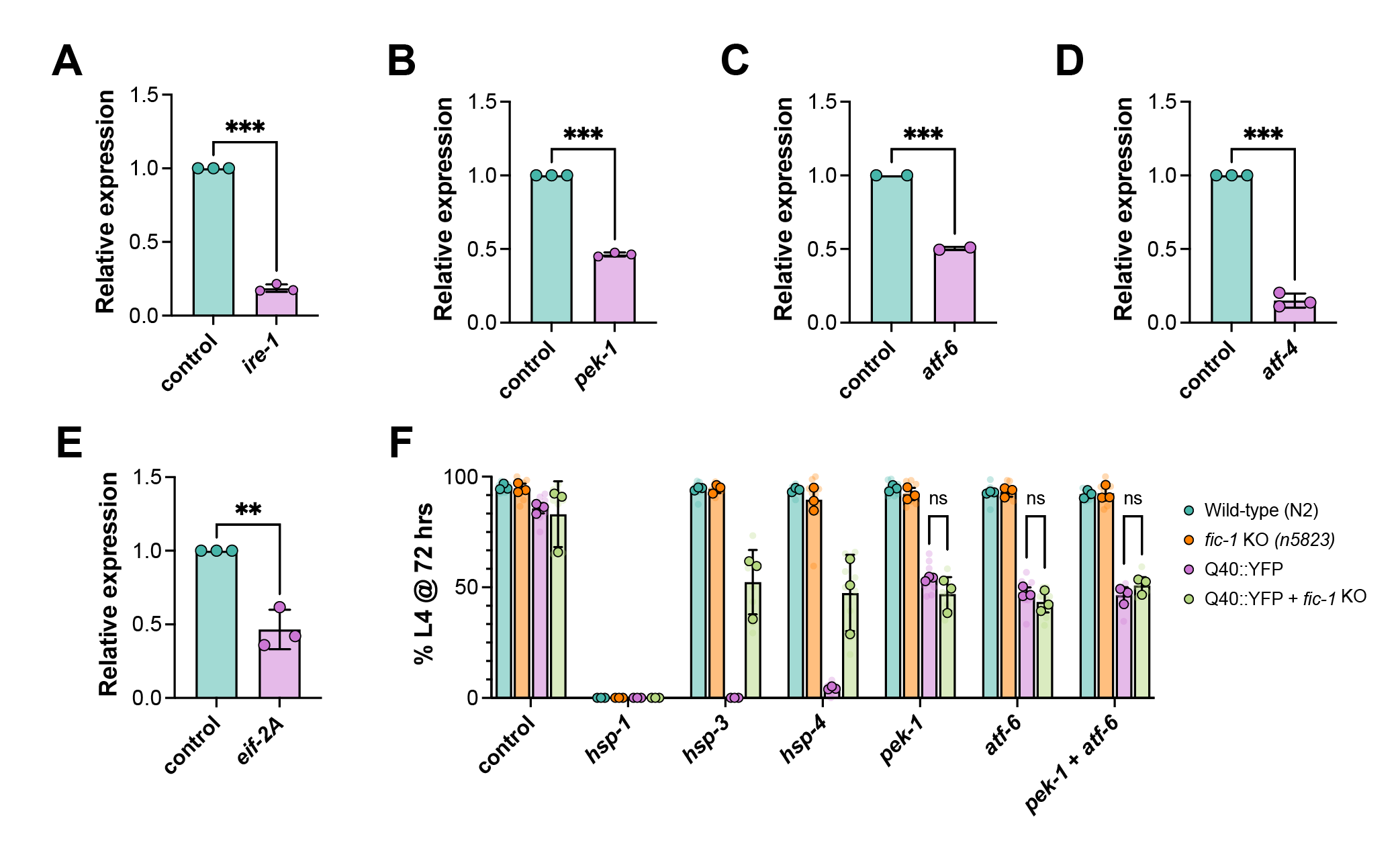

Supplement: S9 Fig — (F) Development assay testing the combinatorial knock-down of pek-1 and atf-6. X-axis indicates RNAi conditions used. Graph depicts the percentage of animals that have reached the L4 stage of larval development when assessed at 72 hours. For (A-E), statistical significance was determined using unpaired T-tests. For (F), statistical significance was assessed using a two-way ANOVA with Tukey’s post-hoc multiple comparisons tests. ***p < 0.001; **p < 0.01; ns = not significant. (TIF) [file pgen.1011723.s009.tif]

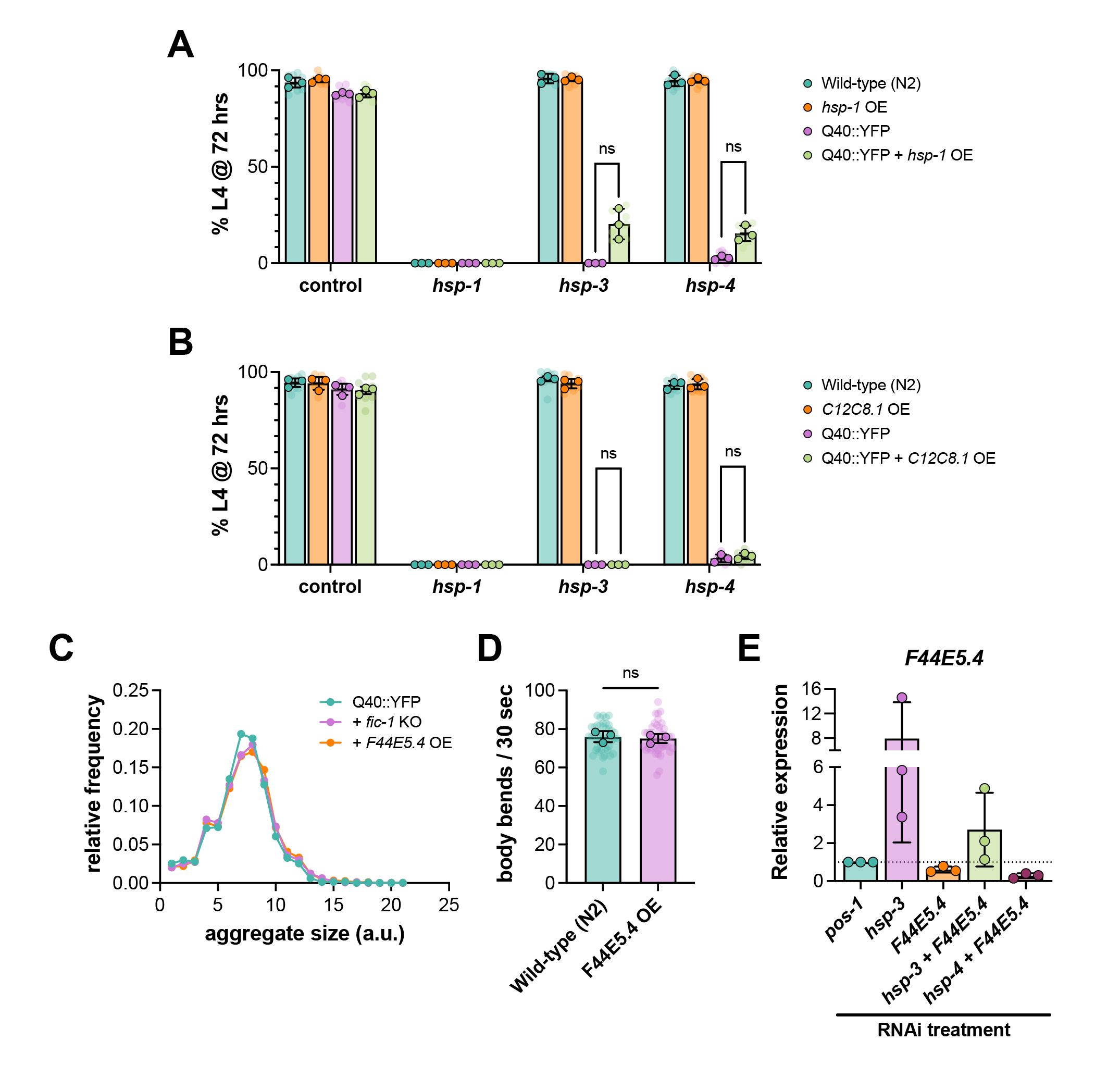

Supplement: S10 Fig — Groupings on X-axes reflect the RNAi condition used. Each graph depicts the percentage of animals that have reached the L4 stage of larval development when assessed at 72 hours. (C) Profile of Q40::YFP puncta size distribution in day 1 adult Q40::YFP, Q40::YFP + fic-1 KO, and Q40::YFP + F44E5.4 OE worms. Bin size = 5 a.u. (D) Thrashing rates of day 1 adult wild-type (N2) and F44E5.4 OE animals. (E) Relative F44E5.4 mRNA expression levels in Q40::YFP + fic-1 KO animals fed the indicated RNAis (X-axis), normalized to control (pos-1). For (A-B), translucent data points reflect technical replicates, while opaque data points depict the average for each biological replicate (n = 3). In (C), at least 50 animals were assessed per genotype. For (D), each translucent data point reflects one individual worm, with at least 45 animals per genotype scored. For (E), each data point represents one biological replicate (n = 3). For (A-B), two-way ANOVA with Tukey’s post-hoc multiple comparisons tests were performed, and in (D) an unpaired T-test was used to assess statistical significance. ***p < 0.001; ns = not significant. (TIF) [file pgen.1011723.s010.tif]
