# Supplementary material for: Loss of FIC-1-mediated AMPylation activates the UPRER and upregulates cytosolic HSP70 chaperones to suppress polyglutamine toxicity
Source: PLoS Genet. 2025 Jun 13;21(6):e1011723. doi: 10.1371/journal.pgen.1011723 (PMC12193957; doi:10.1371/journal.pgen.1011723)
Supplement: S1 Table — (DOCX) [file pgen.1011723.s011.docx]

**Supplementary Table S1.** *C. elegans* strains used in this study

| **Strain name** | **Genotype** | **Source** |
| --- | --- | --- |
| N2 | Wild-type | CGC |
| MT22849 | *fic-1(n5823) IV* | Truttmann et al., 2016 [1] |
| AM138 | *rmIs130[Punc-54::Q24::YFP]* | Morley et al., 2002 [2] |
| AM140 | *rmIs132[Punc-54::Q35::YFP]* | Morley et al., 2002 [2] |
| AM141 | *rmIs133[Punc-54::Q40::YFP]* | Morley et al., 2002 [2] |
| MT24431 | *fic-1(n5823) IV; rmIs130[Punc-54::Q24::YFP]* | Truttmann et al., 2018 [3] |
| MT24425 | *fic-1(n5823) IV; rmIs132[Punc-54::Q35::YFP]* | Truttmann et al., 2018 [3] |
| MT24058 | *fic-1(n5823) IV; rmIs133[Punc-54::Q40::YFP]* | Truttmann et al., 2018 [3] |
| MTX165 | *mtmEx58[Peef-1A.1::HA::F44E5.4; Pmyo-2::GFP]* | This study |
| MTX284 | *mtmIs12[Peef-1A.1::HA::F44E5.4; Pmyo-2::GFP]* | This study |
| MTX162 | *mtmEx55[Peef-1A.1::HA::hsp-1; Pmyo-2::GFP]* | This study |
| MTX315 | *mtmIs9[Peef-1A.1::HA::hsp-1; Pmyo-2::GFP]* | This study |
| MTX168 | *mtmEx61[Peef-1A.1::HA::C12C8.1; Pmyo-2::GFP]* | This study |
| MTX317 | *mtmIs11[Peef-1A.1::HA::C12C8.1; Pmyo-2::GFP]* | This study |
| MTX337 | *fic-1(n5823) IV; mtmIs12; rmIs133* | This study |
| MTX342 | *fic-1(n5823) IV; mtmIs9; rmIs133* | This study |
| MTX335 | *fic-1(n5823) IV; mtmIs11; rmIs133* | This study |

**References**

1. Truttmann MC, Cruz VE, Guo X, Engert C, Schwartz TU, Ploegh HL. The *Caenorhabditis elegans* Protein FIC-1 Is an AMPylase That Covalently Modifies Heat-Shock 70 Family Proteins, Translation Elongation Factors and Histones. PLoS Genet. 2016;12(5):e1006023.

2. Morley JF, Brignull HR, Weyers JJ, Morimoto RI. The threshold for polyglutamine-expansion protein aggregation and cellular toxicity is dynamic and influenced by aging in *Caenorhabditis elegans*. Proc Natl Acad Sci USA. 2002 Aug 6;99(16):10417–22.

3. Truttmann MC, Pincus D, Ploegh HL. Chaperone AMPylation modulates aggregation and toxicity of neurodegenerative disease-associated polypeptides. Proc Natl Acad Sci USA. 2018 29;115(22):E5008–17.
