# Supplementary material for: Loss of FIC-1-mediated AMPylation activates the UPRER and upregulates cytosolic HSP70 chaperones to suppress polyglutamine toxicity
Source: PLoS Genet. 2025 Jun 13;21(6):e1011723. doi: 10.1371/journal.pgen.1011723 (PMC12193957; doi:10.1371/journal.pgen.1011723)
Supplement: S2 Table — (DOCX) [file pgen.1011723.s012.docx]

**Supplementary Table S2.** PCR primers used in this study

| **Primer sequence** | **Description** | **Source** |
| --- | --- | --- |
| CGCTTCGATTTTTTCGCTGACTTCC | *fic-1(n5823)* genotyping primer | Truttmann et al., 2016 [1] |
| CTCGTACAACGCCACTGGTTAGTG | *fic-1(n5823)* genotyping primer | Truttmann et al., 2016 [1] |
| CGTGCAATACTCGAATCAGGAAT | *fic-1(n5823)* genotyping primer | Truttmann et al., 2016 [1] |
| actctagaccgcggttttggcgcgccGCACCTTTGGTCTTTTATTG | *eef-1A.1* into pPD117.01 forward primer | This study |
| taggcggcatAGCCTGCTTTTTTGTACAAAC | *eef-1A.1* into pPD117.01 reverse primer | This study |
| acgtcccagactacgctggcTCTTCATGCAAAGCTATTGGTATCGACC | *F44E5.4* into pMT686 forward primer | This study |
| ttactcattttttctaccggTTTAATCAACTTCCTCAACAGTAGGTCCTTG | *F44E5.4* into pMT686 reverse primer | This study |
| acgtcccagactacgctggcAGTAAGCATAACGCTGTTGGAATCGATTTG | *hsp-1* into pMT686 forward primer | This study |
| ttactcattttttctaccggTTTAGTCGACCTCCTCGATCGTTGG | *hsp-1* into pMT686 reverse primer | This study |
| acgtcccagactacgctggcTCTACATGCAAAGCGATTGGAATCGAC | *C12C8.1* into pMT686 forward primer | This study |
| ttactcattttttctaccggTTTAATCAACTTCCTCTACAGTAGGTCCTTG | *C12C8.1* into pMT686 reverse primer | This study |

**References**

1. Truttmann MC, Cruz VE, Guo X, Engert C, Schwartz TU, Ploegh HL. The *Caenorhabditis elegans* Protein FIC-1 Is an AMPylase That Covalently Modifies Heat-Shock 70 Family Proteins, Translation Elongation Factors and Histones. PLoS Genet. 2016;12(5):e1006023.
