# Supplementary material for: Loss of FIC-1-mediated AMPylation activates the UPRER and upregulates cytosolic HSP70 chaperones to suppress polyglutamine toxicity
Source: PLoS Genet. 2025 Jun 13;21(6):e1011723. doi: 10.1371/journal.pgen.1011723 (PMC12193957; doi:10.1371/journal.pgen.1011723)
Supplement: S3 Table — (DOCX) [file pgen.1011723.s013.docx]

**Supplementary Table S3.** RNAi clones used in this study

| **Target gene** | **Source library** |
| --- | --- |
| Empty vector (pL440) | Fire Lab Vector Kit (Addgene #1654) |
| *pos-1* | *C. elegans* ORFome Library v1.1 [1] |
| *fic-1* | Ahringer *C. elegans* RNAi Feeding Library [2,3] |
| *hsp-1* | Ahringer *C. elegans* RNAi Feeding Library [2,3] |
| *hsp-3* | *C. elegans* ORFome Library v1.1 [1] |
| *hsp-4* | *C. elegans* ORFome Library v1.1 [1] |
| *ire-1* | Ahringer *C. elegans* RNAi Feeding Library [2,3] |
| *xbp-1* | Ahringer *C. elegans* RNAi Feeding Library [2,3] |
| *pek-1* | Ahringer *C. elegans* RNAi Feeding Library [2,3] |
| *atf-6* | Ahringer *C. elegans* RNAi Feeding Library [2,3] |
| *atf-4* | *C. elegans* ORFome Library v1.1 [1] |
| *eif-2A* | Ahringer *C. elegans* RNAi Feeding Library [2,3] |
| *F44E5.4* | *C. elegans* ORFome Library v1.1 [1] |
| *col-135* | Ahringer *C. elegans* RNAi Feeding Library [2,3] |
| *ilys-5* | *C. elegans* ORFome Library v1.1 [1] |
| *lys-10* | *C. elegans* ORFome Library v1.1 [1] |
| *abu-2* | Ahringer *C. elegans* RNAi Feeding Library [2,3] |
| *cpr-8* | *C. elegans* ORFome Library v1.1 [1] |
| *vit-5* | Ahringer *C. elegans* RNAi Feeding Library [2,3] |
| *asp-3* | Ahringer *C. elegans* RNAi Feeding Library [2,3] |
| *clec-53* | Ahringer *C. elegans* RNAi Feeding Library [2,3] |
| *asah-1* | Ahringer *C. elegans* RNAi Feeding Library [2,3] |
| *hsp-16.49* | *C. elegans* ORFome Library v1.1 [1] |
| *skr-3* | Ahringer *C. elegans* RNAi Feeding Library [2,3] |
| *cct-1* | *C. elegans* ORFome Library v1.1 [1] |
| *cct-4* | *C. elegans* ORFome Library v1.1 [1] |
| *sqst-1* | *C. elegans* ORFome Library v1.1 [1] |
| *gst-8* | *C. elegans* ORFome Library v1.1 [1] |
| *ubql-1* | *C. elegans* ORFome Library v1.1 [1] |
| *hsp-16.11* | *C. elegans* ORFome Library v1.1 [1] |
| *hsp-17* | *C. elegans* ORFome Library v1.1 [1] |
| *lec-11* | Ahringer *C. elegans* RNAi Feeding Library [2,3] |
| *dnj-7* | *C. elegans* ORFome Library v1.1 [1] |
| *enpl-1* | *C. elegans* ORFome Library v1.1 [1] |
| *ero-1* | *C. elegans* ORFome Library v1.1 [1] |
| *stc-1* | *C. elegans* ORFome Library v1.1 [1] |
| *ubxn-4* | *C. elegans* ORFome Library v1.1 [1] |

**References**

1. Rual JF, Ceron J, Koreth J, Hao T, Nicot AS, Hirozane-Kishikawa T, et al. Toward Improving *Caenorhabditis elegans* Phenome Mapping With an ORFeome-Based RNAi Library. Genome Res. 2004 Oct;14(10b):2162–8.

2. Fraser AG, Kamath RS, Zipperlen P, Martinez-Campos M, Sohrmann M, Ahringer J. Functional genomic analysis of *C. elegans* chromosome I by systematic RNA interference. Nature. 2000 Nov 16;408(6810):325–30.

3. Kamath RS, Fraser AG, Dong Y, Poulin G, Durbin R, Gotta M, et al. Systematic functional analysis of the *Caenorhabditis elegans* genome using RNAi. Nature. 2003 Jan 16;421(6920):231–7.
