# Supplementary material for: Loss of FIC-1-mediated AMPylation activates the UPRER and upregulates cytosolic HSP70 chaperones to suppress polyglutamine toxicity
Source: PLoS Genet. 2025 Jun 13;21(6):e1011723. doi: 10.1371/journal.pgen.1011723 (PMC12193957; doi:10.1371/journal.pgen.1011723)
Supplement: S4 Table — (DOCX) [file pgen.1011723.s014.docx]

**Supplementary Table S4.** RT-qPCR primers used in this study

| **Target gene** | **Sequence** | **Direction** | **Reference** |
| --- | --- | --- | --- |
| *cdc-42* | CTGCTGGACAGGAAGATTACG | forward | Hoogewijs et al., 2008 [1] |
| *cdc-42* | CTCGGACATTCTCGAATGAAG | reverse | Hoogewijs et al., 2008 [1] |
| *pmp-3* | GTTCCCGTGTTCATCACTCAT | forward | Hoogewijs et al., 2008 [1] |
| *pmp-3* | ACACCGTCGAGAAGCTGTAGA | reverse | Hoogewijs et al., 2008 [1] |
| *pos-1* | GCTTACAAACGCAGTCAGGC | forward | This study |
| *pos-1* | TCTCCCACGAGGGTTCATTG | reverse | This study |
| *atf-4* | AATCGAGCGTCGTTCCAACT | forward | This study |
| *atf-4* | TAGCTGCGAGACGGTTTTGA | reverse | This study |
| *eif-2A* | AGTTTACGGACCAAAGACTGC | forward | This study |
| *eif-2A* | TACGTGCTTTCCATCCGCAA | reverse | This study |
| *hsp-3* | ACCGTCACCATCCAGGTC | forward | Kozlowski et al., 2014 [2] |
| *hsp-3* | TCCGGTGAGGTCGAACTTT | reverse | Kozlowski et al., 2014 [2] |
| *hsp-4* | CAGATGAAAACTCAAATCGCC | forward | Taylor & Dillin, 2013 [3] |
| *hsp-4* | GGTTGCTTCCGAGCCACTCAA | reverse | Taylor & Dillin, 2013 [3] |
| *ire-1* | AAGTGCCGTTTTTGCCGTTT | forward | Urban et al., 2025 [4] |
| *ire-1* | TGAGGACAAGACCATTGGACAG | reverse | Urban et al., 2025 [4] |
| *pek-1* | AAGAAGGTCATCGGGCATGG | forward | Urban et al., 2025 [4] |
| *pek-1* | CCGCAATTCTCTTGACAGCG | reverse | Urban et al., 2025 [4] |
| *atf-6* | CCTACTTTACGGGACCGACG | forward | Urban et al., 2025 [4] |
| *atf-6* | AATCTCCTAAACTCCCGCCG | reverse | Urban et al., 2025 [4] |
| *F44E5.4* | GTCTTCATGCAAAGCTATTGGTATC | forward | Calamini et al., 2011 [5] |
| *F44E5.4* | CGTCGTCCAATCAATCCTTTTGCATC | reverse | Calamini et al., 2011 [5] |

**References**

1. Hoogewijs D, Houthoofd K, Matthijssens F, Vandesompele J, Vanfleteren JR. Selection and validation of a set of reliable reference genes for quantitative sod gene expression analysis in *C. elegans*. BMC Mol Biol. 2008 Jan 22;9:9.

2. Kozlowski L, Garvis S, Bedet C, Palladino F. The *Caenorhabditis elegans* HP1 family protein HPL-2 maintains ER homeostasis through the UPR and hormesis. Proc Natl Acad Sci U S A. 2014 Apr 22;111(16):5956–61.

3. Taylor RC, Dillin A. XBP-1 is a cell-nonautonomous regulator of stress resistance and longevity. Cell. 2013 Jun 20;153(7):1435–47.

4. Urban ND, Lacy SM, Pelt KMV, Abdon B, Mattiola Z, Klaiss A, et al. Functionally diversified BiP orthologs control body growth, reproduction, stress resistance, aging, and ER-Phagy in *Caenorhabditis elegans* [Internet]. bioRxiv; 2025 [cited 2025 Mar 26]. p. 2025.01.14.633073. Available from: https://www.biorxiv.org/content/10.1101/2025.01.14.633073v1

5. Calamini B, Silva MC, Madoux F, Hutt DM, Khanna S, Chalfant MA, et al. Small-molecule proteostasis regulators for protein conformational diseases. Nat Chem Biol. 2011 Dec 25;8(2):185–96.
