# Supplementary material for: Loss of FIC-1-mediated AMPylation activates the UPRER and upregulates cytosolic HSP70 chaperones to suppress polyglutamine toxicity
Source: PLoS Genet. 2025 Jun 13;21(6):e1011723. doi: 10.1371/journal.pgen.1011723 (PMC12193957; doi:10.1371/journal.pgen.1011723)
Supplement: Attachment — Submitted filename: Response_to_Reviewers_Final.docx [file pgen.1011723.s018.docx]

**Rebuttal letter for PLOS Genetics submission PGENETICS-D-24-01488: *Loss of FIC-1-mediated AMPylation activates the UPR^ER^ and upregulates HSP70 chaperones to suppress polyglutamine toxicity***

Dear colleagues,

We would like to thank you all for the insightful comments and helpful suggestions made in response to our initial submission. We also appreciate the journal’s decision to invite us to revise our manuscript according to the reviewers’ suggestions. We have put significant effort into addressing the comments and questions raised by reviewers and are pleased to submit a revised manuscript for your consideration. Below, we address each reviewer’s comments in a point-by-point response.

We believe that our revised figures, additional new data, and written explanations below should resolve any concerns raised by reviewers. We trust that this will result in a positive re-evaluation of our manuscript for publication in PLOS Genetics.

Sincerely,

Matthias Truttmann

**Reviewer 1: Summary and comments**

**Summary:** This manuscript expands on previous findings that fic-1-mediated AMPylation alters polyQ-YFP aggregation dynamics and motility in adult C. elegans. The current work shows that genetic deletion of fic-1 AMPylase rescues developmental arrest, lifespan deficits, and motility impairment in C. elegans with body wall muscle expression of polyQ40 when the worm BiP ortholog hsp-3 or hsp-4 is knocked down, and this rescue depends on intact UPRER sensors ire-1, pek-1, and atf-6. The authors characterize and validate the distinct transcriptomic response of Q40::YFP; fic-1 null worms to hsp-3 or hsp-4 knockdown that appears to facilitate rescue by restoring ER homeostasis. They identify the HSP70 family chaperone F44E5.4 as a gene whose induction depends on intact UPRER sensors and whose overexpression rescues disease polyQ toxicity with hsp-3 or hsp-4 knockdown. This work is impactful in its identification of a suppressor of polyQ toxicity in the context of ER stress that is responsive to AMPylation status, suggesting that modification of AMPylation status or targeting of a single chaperone might be therapeutically useful. However, the paper exhibits limitations in methods and therefore generalizability of these findings, which are made clear in the discussion and limitations sections.

**Major concerns:**

1) This study is heavily dependent upon RNAi methodology and body wall muscle expression of polyQ.

*We agree with Reviewer #1 that our study primarily utilizes RNAi to reduce target gene expression. We have thus invested significant effort into developing an alternative method to selectively deplete or delete hsp-3 and hsp-4 in the Q40::YFP model, albeit without success. Due to the lethality of losing either hsp-3 or hsp-4 in the Q40::YFP background, we have been unable to recapitulate our RNAi results using available hsp-3/4 null alleles. In an attempt to circumvent this issue, our lab then developed auxin-inducible degron (AID) strains to selectively deplete HSP-3 and HSP-4. Using CRISPR/Cas9, we introduced an AID sequence at the C-terminus of the hsp-3 and hsp-4 genes, upstream of the terminal KDEL motifs. The resulting worm strains expressing HSP-3::AID::3xFLAG::KDEL and HSP-4::AID::3xFLAG::KDEL were healthy and expressed the modified genes at similar levels to the unmodified endogenous copies in wildtype animals. We then crossed these strains into full body and tissue-specific TIR1 driver strains. TIR1 is an adaptor protein required to present AID-tagged proteins to the proteosome for degradation. Unfortunately, however, none of the existing TIR1-driver strains we were able to source*^1^ *and test enabled degradation of AID-tagged HSP-3 or HSP-4 The likely reason for this is that currently available TIR1 strains are limited to TIR1 expression in the cytosol and the nucleus, precluding the degradation of ER-resident substrates. It remains further to be shown that the AID-TIR1 technology is applicable to ER-resident proteins. Based on our results, we are now contemplating to develop a new degron system specifically for ER-resident proteins, following the strategy used in human cells and described in Sharninghausen et al.*^2^*; a project which will keep us busy for months to come. Despite being unsuccessful in utilizing complementary methodology, we remain convinced that the validated RNAi-base approaches we took to specifically knockdown hsp-3 and hsp-4 suffice to draw the presented conclusions.*

2) The specificity of RNAi here is not demonstrated for hsp-3 and hsp-4. These are highly similar orthologs and the claim that RNAi treatment of each is specific for one and not the other of hsp-3/4 must be demonstrated. For example, does hsp-3 RNAi treatment impact hsp-4 transcript levels and visa versa.

*We appreciate the Reviewer mentioning the specificities of these RNAis as an essential control which will add to the validity and reproducibility of this manuscript. We have addressed this point through re-analysis of our RNAseq data and complimented these findings with qPCR studies, all of which are shown in* ***Supplementary Fig 5A-E****. In a differential expression (DE) analysis of Q40::YFP; fic-1 KO animals on each RNAi condition, we find that hsp-3 knock-down results in a non-significant slight increase in hsp-4 transcripts. This finding is reflected in graphs of the raw FPKM values, validated by qPCR, and has also been published previously*^3^*. On hsp-4 RNAi, we find no significant difference in hsp-3 expression by DE analysis. However, both the FPKM values and qPCR suggest a slight decrease in hsp-3 expression. Given that changes in hsp-3 expression have been shown to impact hsp-4 expression levels*^3^*, the observed changes may reflect compensatory up/down regulation rather than RNAi-mediated off-targeting.*

3) Including validation of at least the main findings using a neuronal polyQ would be more disease relevant and increase the impact of this work. An ideal experiment would be to recapitulate the RNAi findings from muscle instead in neurons. This could be accomplished using existing hsp-3 and/or hsp-4 loss of function alleles.

*We appreciate this suggestion from the Reviewer and do agree that extending these findings into a more disease-relevant model could increase the impact of this work. However, we do not claim in the paper that expressing polyQ::YFP in muscle cells has any explicit disease link; rather, we see and introduce it as a model to trigger proteotoxic stress in cells. We assume that the proposed move into neurons is motivated by the abundance of polyQ repeat disorders affecting this cell type. While we appreciate this suggestion, a focus on neurons is not required to determine how the ER-resident enzyme, FIC-1, helps mitigate proteotoxic stress in the cytosol. Furthermore, there are technical limitations that prevented us from performing the proposed experiments: First, as for strains expressing polyQ repeat proteins in muscle cells, our attempts to cross hsp-3 and hsp-4 null alleles into a neuronal polyQ(86)::YFP proved impossible. This indicates that losing either hsp-3 or hsp-4 while expressing polyQ(86)::YFP in neurons is lethal, which is consistent with what we observed in muscle cells. Second, neurons are insensitive to RNAi in C. elegans. To render neurons RNAi sensitive, a sid-1 transgene controlled by a neuronal promotor such as rgef-1 or unc-119 needs to be introduced. We attempted to cross two distinct neuronal sid-1 alleles from strains TU3311 and MAH677 into a neuronal polyQ(86)::YFP strain but to no avail.*

4) If the claim is that fic-1 is beneficial in the context of ER stress, why not induce ER stress genetically using xbp-1s transgenes or pharmacologically as an orthogonal method to RNAi?

*We chose to destabilize the ER proteome through selective depletion of HSP-3/HSP-4 because both were already known targets of FIC-1-mediated AMPylation. Our prior work had shown minimal impact of increased/decreased AMPylation in acute and chronic ER stress assays in the absence of polyQs*^4^*. Given our interest in FIC-1’s role in cellular proteostasis, the existence of two BiP orthologs in C. elegans presented us with the unique opportunity to target two known FIC-1 substrates and study the resulting effects without incurring the lethal effects of knocking-down an essential chaperone that is present as a single copy in most other organisms*^5^*.*

*However, we agree with reviewer #1 that the induction of ER stress using genetic or pharmacological approaches would complement our work. We thus performed additional experiments using the pharmacological ER stressors tunicamycin and thapsigargin in development assays (****Supplementary Fig. S3C-D****). We found that fic-1 deficiency significantly buffered developmental resilience to tunicamycin stress in the absence of polyQ proteins, a result consistent with published work*^4^*. This benefit was negated in the presence of polyQ proteins. We also found that treatment with both tunicamycin (2.5 μg/mL & 5 μg/mL) and thapsigargin (5uM) significantly reduced the survival of polyQ; fic-1 KO animals compared to fic-1 KO animals. This provides another example of how polyQ proteotoxicity impacts ER proteostasis and further supports the model that fic-1 may act as a rheostat, as proposed in studies of its human ortholog, FICD*^6^*. Depending on the acuteness and type of ER stress, the loss of fic-1-mediated protein AMPylation may either benefit or destabilize proteostasis. Our results clearly show that fic-1 deficiency is not universally beneficial in the face of ER stress, but rather context-specific. We address this point now in the discussion section as follows:*

*“Our finding that fic-1 deficiency buffers against tunicamycin stress in the absence, but not the presence, of polyQ proteins (****Supplementary Fig. S3C****) underscores the recently proposed notion that FIC-1/FICD serves to act as a rheostat, fine-tuning the response to ER stress*^6^*. While we find that the loss of FIC-1-mediated AMPylation is protective in the context of polyQ toxicity, it is not universally beneficial – for example, we previously reported that increased AMPylation is protective in a C. elegans model of amyloid-β toxicity*^7^*. As such, the effects of fic-1/Ficd deficiency in the face of ER stress are likely dependent on the stressor, duration of stress, and the longevity of the cell types involved.”*

5) Whole organism overexpression of F44E5.4 precludes the reader from determining whether the phenotypic rescue is cell autonomous or non-cell autonomous since polyQ is being expressed in body wall muscle. Tissue specific rescue would be better.

*We agree that the whole-body over-expression approach used to verify the beneficial effects of F44E5.4 upregulation precludes further mechanistic insights into the cell types or tissues required for the rescue. We have added a statement to the “Limitations of this study” section highlighting this point to the reader.*

**Minor concerns:**

1) The language parsing the difference between hsp-3 and hsp-4 as BiP orthologs seems unclear. Please include explanation that hsp-3 as constitutive, hsp-4 as the UPR inducible molecular chaperone.

*We have added language to the introduction section to better explain the functions of both BiP orthologs. It reads: “These two ER-resident paralogs, HSP-3 and HSP-4, are thought to have arisen through a gene duplication event*^8,9^*, While HSP-3 is expressed constitutively at high levels throughout the worm, HSP-4 exhibits lower basal expression and is highly upregulated in response to stress*^3^*.”*

2) “aggregate” is used in the main text but “puncta” is used in methods and figure legends. Would be prudent to clarify the semantic difference (i.e. “aggregate” suggests insoluble, but solubility assay not performed in this paper.

*We agree with the Reviewer and have replaced the word “aggregate” with “puncta” when used in reference to the C. elegans model or experimental results.*

3) Lines 345-346 “Combinatorial knock-down of fic-1 + hsp-3 or fic-1 + hsp-4 in wild-type or Q40::YFP animals recapitulated this rescue (Supplementary Fig. S1C), suggesting a cell-autonomous, rather than a neuron-involving cell non-autonomous, mechanism.” Requires clarification or should be preceded by explanation of the limitation of RNAi on neurons. Just because the combinatorial RNAi knockdown recapitulated the rescue does not necessarily mean that the rescue is cell-autonomous.

*We agree with this comment. We have added clarifying language on the limitations of RNAi in C. elegans neurons. We have also softened the statement to reflect that, while the rescue is likely not driven primarily through neurons, it is not necessarily fully cell-autonomous. The statement now reads:*

*“Given the limited effect of RNAi in C. elegans neurons due to a lack of the dsRNA transporter, SID-1*^10^*, we posit that the rescue observed here is predominantly non-neuronal.”*

4) Fig.1B-C uses an antibody for Thr AMP but not Ser AMP. Is the reader to assume that Thr AMP and Ser AMP sites are AMPylated at similar rates?

*We used the Thr-AMP antibody to detect HSP-3/HSP-4 AMPylation as this is the only antibody we have found that reliably detects endogenous protein AMPylation in our hands. To the best of our knowledge, no comparable antibody against Ser-AMP sites exists.*

*Prompted by this question, we performed in vitro AMPylation reactions with recombinant FIC-1(E274G) and either HSP-3 or HSP-4 and submitted them for mass spectrometry. This new data is shown in* ***Supplementary Fig. S3A-B****. We found that HSP-3 is AMPylated on T194, while HSP-4 was AMPylated on T368, T455, and T576. We did not detect any other modified residues. As such, serine AMPylation is unlikely to play a role in HSP-3/HSP-4 regulation.*

5) Does fic-1 deletion rescue polyQ tracts longer than 40 upon hsp-3/4 knockdown? The Q40 is a relatively mild phenotype compared to longer expansions.

*We appreciate this question raised by Reviewer #1 and invested considerable effort to test the effect of fic-1 loss in strains expressing longer polyQ tracts. We initially contacted the lab of Rick Morimoto lab, who generated the Q40::YFP model employed in this study, to inquire about their previously published Q64::YFP and Q82::YFP strains*^11^*. Unfortunately, we were informed that both strains were highly unstable and unable to be recovered from freezing, and as such have been lost. Instead, we were able to source a strain expressing muscular Q82::YFP as an extra-chromosomal array, shared with us by the lab of Ellen Nollen. However, the Q82::YFP-encoding array is highly unstable, with animals exhibiting marked transgene silencing when we attempted to test them in our development assay set-up (see images below). We reasoned that the high degree of toxicity incurred by Q82::YFP expression may have resulted in partial silencing of the extra-chromosomal array in some animals, as has been reported previously*^12,13^*. This uneven Q82::YFP expression pattern made it impossible for us to obtain reliable and reproducible data, even in untreated Q82::YFP animals.*

**Above**: Fluorescent image showing variable and downregulated expression of the Q82::YFP extrachromosomal array in a population of adult *C. elegans*. Scale bar = 100um.

6) F44E5.4 and C12C8. are both orthologs of HSPA6 and HSPA7 according to WormBase. Could the authors comment on the specificity of F44E5.4 rescue and how is this translatable if the orthologs are the same?

*This is an interesting question. Indeed, WormBase links both genes to HSPA6 and HSPA7. However, this linkage is solely based on automated sequence homology comparisons and not on any functional data, nor is it necessarily human curated. HSPA6 and HSPA7 share more than 90% sequence identity*^14^*; it is thus not surprising that homology models will highlight resemblance to both genes. Further, it is important to note that despite sharing homology to similar human orthologs and one another, some related chaperones have evolved very distinct functions. In a parallel study, disclosed on bioRxiv*^15^*, we recently demonstrated that HSP-3 and HSP-4 exhibit both shared and distinct molecular functions during different types of ER stress, despite sharing >80% sequence homology. Our finding that F44E5.4, but not C12C8.1 or hsp-1 can improve development of polyQ-expressing worms depleted of hsp-3 represents yet another example where each Hsp70 family chaperone, regardless of its homology to other family members, may well have acquired unique functions that make it irreplaceable. As disclosed in the hsp-3/hsp-4 article on bioRxiv, we are currently in the process of translating and validating some of these findings in human cells.*

7) Please clarify whether there are other AMPylation targets besides BiP, i.e. does fic-1 deletion have other effects? If possible, more clearly state the molecular steps from fic-1 loss to UPR activation via BiP

*Our lab has previously identified additional FIC-1 targets beyond HSP-3 and HSP-4, including translation elongation factors (EEF-1A.2, EEF-1G, EEF-2), histone H3, and the HSP70 family chaperone HSP-1 (ortholog of human HSC70)*^4^*. However, unlike HSP-3 and HSP-4, these targets have not yet been shown to be AMPylated in vivo. We added a statement to the limitations section to highlight this point better:*

*“Lastly, we have previously identified additional targets of FIC-1-mediated AMPylation in vitro beyond HSP-3 and HSP-4, including translation elongation factors (EEF-1A.2, EEF-1G, EEF-2), histone H3, and the HSP70 family chaperone HSP-1 (ortholog of human HSC70)*^4^*. While, unlike HSP-3 and HSP-4, these targets have not yet been shown to be AMPylated in vivo, we recognize that fic-1 deletion has the likely potential to impact AMPylation targets that have yet to be characterized.”*

*With regards to UPR activation, the predominant model in the field states that, in the absence of ER stress, FIC-1 AMPylates HSP-3/4 (BiP), inactivating these chaperones*^16,17^*. During ER stress, FIC-1 functions to deAMPylate HSP-3/4, increasing the supply of available chaperones*^18^*. The exact steps linking FIC-1 deAMPylation activity (or fic-1 loss) to UPR activation have yet to be fully delineated.*

8) Introduction (line 48-51) “One prominent example is the endoplasmic reticulum (ER)-resident HSP70 chaperone, BiP, which can trigger the ER’s unfolded protein response (UPRER) and downstream stress-response signaling through activation of UPRER sensors IRE1 and PERK” should also include ATF6

*Edited as suggested.*

9) Introduction (line 53-54) “However, the consequences of such a strategy, which include the potential to fuel e.g. cancer cells” remove “e.g.”

*Edited as suggested.*

10) Discussion: “multi-facetted” should be “multifaceted” (line 590); instead of “sustained fic-1 activity” would suggest “intact” (line 594); should be “complementary” (line 640)

*Edited as suggested.*

11) A more thorough discussion of similarities and differences between mammalian & worm UPR would be helpful for the discussion. Perhaps, mention lack of CHOP downstream of UPR in worms in discussion (lines 632-651).

*We appreciate this suggestion from the Reviewer and have expanded our discussion section around this topic. The following statements were added/edited:*

*“While the UPR^ER^ is highly conserved across species, one notable difference in C. elegans is the lack of an ortholog to mammalian C/EBP homologous protein (CHOP)*^19,20^*, a transcription factor that is activated downstream of the UPR^ER^ to mediate ER stress-induced apoptosis. As such, further studies in physiologically-relevant cell types (e.g. neurons) and higher-order mammalian models of neurodegeneration are needed to determine the extent to which our findings of a UPR^ER^-dependent protective effect from fic-1 loss are generalizable.”*

**Reviewer 2: Summary and comments**

**Summary:** This study investigates how the AMPylase enzyme FIC-1 interferes with protein homeostasis maintenance during pathologic protein aggregation. Protein AMPylation is an important post-translational modification to regulate the activity of Hsp70 chaperones, in particular for the endoplasmic reticulum Hsp70 BiP, which serves a master regulator of the unfolded protein response (UPR). Many studies, including ones from the senior of author here, have shown how FIC-1 mediated AMPylation is important for modulating the aggregation of neurodegenerative diseases-associated proteins and that FIC-1 regulates UPR signaling. This study now provides further insights how FIC-1 dampens the UPR under proteotoxic stress and is detrimental to organismal development. In a C. elegans model of proteotoxicity from combined polyQ repeat expansion and knockdown of the BiP orthologs hsp3 and 4, the author find that FIC-1 knockout relieves larval arrest. Using transcriptomics and genetic approaches, they establish that the FIC-1 knockout enhances signaling through multiple UPR pathways. Overall, this study is carried out rigorously in most part, the conclusions are supported by the experimental data, and the manuscript is well written. Some limitations are pointed out. The detailed characterization of UPR signaling under hsp3 vs. hsp4 knockdown and discovery of a specific cytosolic chaperone, F44E5.4, which is regulated by the UPR and suppressed by FIC-1, is especially interesting, highlighting adaptability of the UPR and existence of specialized signaling programs. This study should be of interest to a broad readership interested in protein aggregation diseases, proteostasis and stress signaling. The findings have interesting wider implications on whether FIC-1 inhibition can enhance physiology under less construed proteotoxic stress conditions in other organisms.

**Comments:**

1) To cause proteotoxic stress in the Q40:YFP strain, the authors deplete hsp3 or hsp4, which are directly connected to FIC-1 signaling as they are the predominate AMPylation targets. One open question is whether FIC-1 knockout can protect from other proteotoxic stressors through a similar mechanism. I am sympathetic that this is a larger question that may be better suited for a follow up manuscript, but I am curious whether the authors have compared other ER stressors in the Q40:YFP FIC-1 KO vs. Q40:YFP backgrounds.

*We appreciate this question from Reviewer #2, as we have also been interested in how the loss of AMPylation impacts the organismal response to other proteotoxic stressors in the context of polyQ aggregation. To answer this question, we performed development assays in the presence of two compounds known to induce ER stress through distinct mechanisms – tunicamycin (Tm), and thapsigargin (Tg). This new data is shown in* ***Supplementary Fig. S3C-D****.*

*In the absence of polyQs, we observed that fic-1(n5823) animals were less susceptible to the impact of Tm on larval development at higher concentrations. In the presence of polyQs, however, we saw an interesting trend towards increased Tm sensitivity in the fic-1 KO background While Tg treatment did not have a significant impact on worm development in the absence of polyQs, we observed a similar trend for Q40::YFP; fic-1 KO animals at the 5μM concentration. We thus hypothesize that loss of fic-1 results in a hormesis-like effect where, in the presence of one stressor (aggregating proteins, chemical stressors), less AMPylation is beneficial, but becomes detrimental when multiple proteotoxic insults are combined. An alternative explanation could be that fic-1, similar to its mammalian ortholog, FICD, may act as a rheostat: depending on the amount and type of stress, fic-1 deficiency may benefit or exacerbate stress-related phenotypes.*

2) In Fig. 1B (and S1A-B), the residual AMPylation in the hsp4 knockout is stronger than in the hsp3 knockout (especially in WT N2). Does this reflect relative expression differences of hsp3 and hsp4, or are the two proteins AMPylated to different levels? This may have important ramifications for which of the BiP orthologs is more sensitive to regulation by FIC-1 and important for activating the specific UPR-ER signaling dependencies identified later. Maybe the authors could use the RNA-Seq data to (crudely) compare the relative hsp3/4 expression to tease these possibilities apart.

*At baseline, us and other labs have reported that hsp-3 is constitutively expressed at high levels throughout the worm, while hsp-4 is expressed at much lower levels and is restricted to specific tissues such as the spermatheca*^15,21^*. As suggested by the reviewer, we graphed the FPKM values for both transcripts from our RNAseq data and found that they are in agreement with this finding (see* ***Supplementary Fig. S5C****). Given the significantly higher expression of hsp-3, we suggest that the stronger Thr-AMP signal observed in the hsp-4 knock-down condition is reflective of this higher expression level. Additionally, we performed mass spec of AMPylated recombinant HSP-3 and HSP-4 (see response to Reviewer #1, point #4, above). From this, we identified 1 AMPylated residue on HSP-3 and 3 AMPylated residues on HSP-4. We introduce these new findings in the main text as follows:*

*“This signal partially decreased upon knock-down of hsp-3 or hsp-4, consistent with AMPylation of both chaperones. To corroborate this approach, we additionally performed in vitro AMPylation reactions with recombinant FIC-1(E274G) and HSP-3 or HSP-4 and submitted these samples for analysis by mass spectrometry. This revealed one AMPylated residue on HSP-3 (T194), and three AMPylated residues on HSP-4 (T368, T455, T576) (****Supplementary Fig. S3A-B****).”*

3) Another questions that remains unaddressed is an explanation for what causes the larval arrest if not the polyQ aggregates themselves? It is somewhat surprising that the levels of polyQ aggregation are not reduced under the rescue in the FIC-1 hsp3/4 double knockout. That being said, it is likely that other proteotoxic species, such as smaller soluble aggregate species, are more detrimental. At minimum, the authors should expand the discussion around this point.

*We appreciate the Reviewer raising this point and have expanded the discussion section with possible explanations for this phenomenon as follows:*

*“We found that despite the dramatic rescue imparted by the loss of fic-1, we did not observe a reduction in polyQ levels or the number of polyQ puncta. One possibility is that the absence of fic-1 results in changes to polyQ solubility or sequestration, as soluble polyQ intermediates are often considered the primary drivers of cytotoxicity. Further studies into the impact of AMPylation on polyQ protein aggregation, including proteins known to be involved in the interplay between polyQ toxicity and ER stress, such as p97/VCP, will be essential for defining the direct impact of AMPylation on these proteotoxic species.”*

4) Does the hsp3 and hsp4 knockdown lead to UPR-ER activation in the absence of polyQ expression? What genes are commonly upregulated in the hsp3 and hsp4 KDs regardless of the strain (center of the Venn diagram in Fig. 3C & F)?

*In the absence of polyQs, we observe a somewhat similar trend in gene activation – hsp-3 knock-down results in the upregulation of predominantly protein folding-related genes, while hsp-4 knock-down activates a more UPR^ER^-specific transcriptional response. This is exemplified when we look at the GO terms for commonly upregulated genes, now visualized in* ***Supplemental Fig. S5F-G****. We have also included tables containing these gene lists grouped by GO term in the Supplementary Information section.*

5) Line 488-489: Can the authors clarify what implicated the IRE-1 and PEK-1 branch specifically in the Gene Ontology. This is not clear from the panels.

*We appreciate the Reviewer bringing this to our attention. We have modified the statement to refer to UPR^ER^ signaling more broadly and have corrected the figure panels referenced. It now reads “Given our data implicating UPR^ER^ signaling in the response to polyQ protein toxicity in the absence of fic-1 (Fig. 3E, 3G)…”.*

6) For the heatmaps of prioritized genes in Fig 3I-J, the authors should also show the other strain conditions to better highlight that the activation is specific to the Q40:YFP; fic-1 KO condition.

*We extracted the upregulated genes specific to wild-type (N2) and fic-1 KO under both hsp-3 and hsp-4 RNAi conditions and have generated the requested heatmaps depicting all genes. Gene ontology analysis of the identified genes did not reveal any specific pattern with regards to biological process, cellular compartment, or molecular function regardless of strain or RNAi condition. To reflect this, we have inserted a statement summarizing the findings and included the heatmaps in* ***Supplementary Fig. S8A-D****.*

7) The authors observed strong induction of both F44E5.4 and F44E5.5, but only tested F44E5.4 for suppression of larval arrest. Did they also investigate F44E5.5. Are these proteins paralogs? How closely related to other Hsp70 chaperones are these?

*F44E5.4 and F44E5.5 are paralogs and share 100% amino acid identity*^9^*. As such, it is sufficient to only clone one gene and test it in transgenic over-expression strains. These chaperones are most closely related to C12C8.1 and hsp-1 (87% and 69% sequence identity), followed by more distantly related HSP70-family chaperones (hsp-3: 57%, hsp-4: 59%, hsp-6: 48%)*^22^*.*

8) What is the knockdown efficiency of F44E5.4 in the combinatorial knockdown experiments? Could incomplete knockdown explain the relatively weak reduction in the L4 rescue?

*We thank Reviewer #2 for this question. To address this point, we extracted RNA from L4 Q40::YFP; fic-1 KO worms grown under the same RNAi conditions used in Fig. 5D (excluding hsp-1) and assessed F44E5.4 transcript levels via qPCR, as shown in* ***Supplementary Fig. S10E****. As expected, we observed a strong upregulation of F44E5.4 on hsp-3 RNAi. Under the combinatorial knock-down condition with hsp-3 (hsp-3 + F44E5.4), F44E5.4 RNAi reduced transcript levels upregulated upon hsp-3 knock-down (8-fold vs. 2.7-fold), but F44E5.4 expression remained elevated compared to the control condition. In combination with hsp-4 RNAi, F44E5.4 RNAi significantly depleted F44E5.4 transcripts. Based on these results, we speculate that the weak rescue effect observed in our development assay is likely due, in part, to the inability of F44E5.4 RNAi to fully suppress F44E5.4 upregulation in response to hsp-3 knock-down.*

9) I suggest integrating the control qPCR from Fig. S7E to the main text panel Fig. 5E for easier comparison. This will make it clearer that the ire-1 and atf-6 RNAi only incompletely attenuate the F44E5.4 activation.

*We appreciate this suggestion and have made the recommended changes. The control qPCR has been removed from the supplement integrated into main text panel Fig. 5E.*

**Reviewer 3: Summary and comments**

**Summary:** In this study by van Pelt et al., the authors investigate how loss of FIC-1, an ampylase that removes AMP residues from posttranslationaly modified proteins, influences proteotoxicity caused by polyQ40 expression using C. elegans as a disease model. They show that depletion of the ER Hsp70 orthologues in C. elegans, hsp-3 or hsp-4, leads to larval arrest in the PolyQ40 disease model that can be rescued by deletion of fic-1. The authors went to investigate the underlying reason for this, and find that all three UPR ER stress pathways are activated. Interestingly, fic-1 deletion during hsp-3 RNAi leads to an upregulation of the cytosolic Hsp70 isoform F44E5.4, that acts as a critical suppressor of PolyQ toxicity. All in all, the study is well controlled and has a logical flow. It provides important and new insights into the mode and mechanism of why fic-1 deletion has beneficial effects in protein folding diseases which will be a significant addition to our knowledge in the field.

**Major comments:**

1) The study uses the Q40::YFP expressed in bodywall muscle to investigate effects of fic-1 ko. IS this specific for PolyQ or are other disease models similarly impacted? For example amyloid beta (1-42) expressed in the muscle?

*Our lab has previously published data showing that expression of the constitutively-active AMPylase, FIC-1(E274G), increased the lifespan of C. elegans expressing amyloid-β_1-42_ in muscle cells by enhancing Aβ aggregation into larger, insoluble aggregates*^7^*. In contrast, loss of fic-1 had no impact on Aβ toxicity or aggregation in this model. Similar results were obtained when studying the impact of FIC-1 activity on a-synuclein toxicity in C. elegans*^7^*. As such, changes in AMPylation have different effects depending on the disease-associated protein involved.*

2) One point I found confusing is that the authors say fic-1 k.o. activates all three branches of UPR-ER signaling during development to combat misfolded proteins. But the read-out for statement is the ability of Q40 expressing C. elegans depleted for fic-1 to develop into L4s during RNAi of ire-1, xbp-1, pek-1, atf-6. It is not solely dependent on the upregulation F44E5.4, since pek-1 RNAi in combination with hsp-3 RNAi does not lead to a reduction of F44E5.4. What are other targets controlled by these transcription factors that could potentially contribute to the beneficial effect. This can also be addressed textually.

*While F44E5.4 upregulation is sufficient to suppress polyQ toxicity in our model, it is likely that additional downstream targets activated in response to hsp-3 knock-down contribute to this effect. Looking at the gene categories upregulated in our RNAseq study, we see a notable oxidative stress signature in the form of several glutathione-S-transferase (gst-) enzymes upon hsp-3 knock-down. Interestingly, previous studies have identified oxidative stress genes as pek-1 targets during ER stress*^23^*. Further, human cells deficient in the downstream PERK mediator, ATF4, showed impairments in glutathione biosynthesis and oxidative stress resistance*^24^*. In the case of hsp-4 knock-down, the upregulation of numerous ERAD-associated genes is likely also beneficial. In C. elegans, the IRE-1/XBP-1 signaling pathway has been shown to be essential for activating ERAD genes in response to ER stress*^25^*. Taken together, multiple gene programs outside of the F44E5.4 rescue likely contribute to the observed detoxification of polyQ puncta in our model, orchestrated by overlapping efforts from all three UPR^ER^ stress sensors.*

*We have expanded the discussion section around this point to highlight that other activated genes likely contribute to the observed stress response, using the examples above.*

**Minor comments:**

1) Figure 4. Provide titles for Figures 4A, 4B, 4C to increase clarity.

*Edited as suggested. Each panel of Figure 4 now includes a title highlighting which UPR^ER^-related genes are being tested. For example, the title of Figure 4A reads: “UPR^ER^ mediators: ire-1 and xbp-1”.*

2) It is not clear why pos-1 RNAi is used as control (p13, line 352) and Figure S1. This needs clarification in the text.

*The pos-1 gene encodes a cytoplasmic CCCH zinc-finger protein (POS-1) that is essential for cell fate determination during C. elegans early embryogenesis*^26,27^*, but dispensable in adult animals*^28^*. Animals fed bacteria expressing dsRNAs against pos-1 from hatching are unaffected, but produce non-viable progeny. As such, we used pos-1 RNAi in this study to prevent the hatching of unwanted offspring that may interfere with experiments*^29^*. This approach allowed us to avoid the use of 5-fluoro-2’-deoxyuridine (FUdR), which has been reported to perturb multiple aspects of worm physiology, including the heat shock response and protein aggregation*^30,31^*.*

*We have added a statement similar to the above in the Materials and methods section of the manuscript (see RNA interference (RNAi) feeding sub-heading) to clarify this point.*

**References**

1.	Zhang, L., Ward, J.D., Cheng, Z., and Dernburg, A.F. (2015). The auxin-inducible degradation (AID) system enables versatile conditional protein depletion in C. elegans. Development *142*, 4374–4384. https://doi.org/10.1242/dev.129635.

2.	Sharninghausen, R., Hwang, J., Dennison, D.D., and Baldridge, R.D. (2024). Identification of ERAD-dependent degrons for the endoplasmic reticulum lumen. eLife *12*. https://doi.org/10.7554/eLife.89606.2.

3.	Kapulkin, W.J., Kapulkin, V., Hiester, B.G., and Link, C.D. (2005). Compensatory regulation among ER chaperones in C. elegans. FEBS Lett *579*, 3063–3068. https://doi.org/10.1016/j.febslet.2005.04.062.

4.	Truttmann, M.C., Cruz, V.E., Guo, X., Engert, C., Schwartz, T.U., and Ploegh, H.L. (2016). The Caenorhabditis elegans Protein FIC-1 Is an AMPylase That Covalently Modifies Heat-Shock 70 Family Proteins, Translation Elongation Factors and Histones. PLoS Genet. *12*, e1006023. https://doi.org/10.1371/journal.pgen.1006023.

5.	Wang, M., Wey, S., Zhang, Y., Ye, R., and Lee, A.S. (2009). Role of the Unfolded Protein Response Regulator GRP78/BiP in Development, Cancer, and Neurological Disorders. Antioxid Redox Signal *11*, 2307–2316. https://doi.org/10.1089/ars.2009.2485.

6.	Casey, A.K., Gray, H.F., Chimalapati, S., Hernandez, G., Moehlman, A.T., Stewart, N., Fields, H.A., Gulen, B., Servage, K.A., Stefanius, K., et al. (2022). Fic-mediated AMPylation tempers the unfolded protein response during physiological stress. Proc Natl Acad Sci U S A *119*, e2208317119. https://doi.org/10.1073/pnas.2208317119.

7.	Truttmann, M.C., Pincus, D., and Ploegh, H.L. (2018). Chaperone AMPylation modulates aggregation and toxicity of neurodegenerative disease-associated polypeptides. Proc. Natl. Acad. Sci. U.S.A. *115*, E5008–E5017. https://doi.org/10.1073/pnas.1801989115.

8.	Snutch, T.P., Heschl, M.F.P., and Baillie, D.L. (1988). The *Caenorhabditis elegans hsp70* gene family: a molecular genetic characterization. Gene *64*, 241–255. https://doi.org/10.1016/0378-1119(88)90339-3.

9.	Hasnain, P., and Kaneko, G. Phylogenetic annotation of Caenorhabditis elegans heat shock protein 70 genes. MicroPubl Biol *2022*, 10.17912/micropub.biology.000633. https://doi.org/10.17912/micropub.biology.000633.

10.	Calixto, A., Chelur, D., Topalidou, I., Chen, X., and Chalfie, M. (2010). Enhanced neuronal RNAi in C. elegans using SID-1. Nat Methods *7*, 554–559. https://doi.org/10.1038/nmeth.1463.

11.	Morley, J.F., Brignull, H.R., Weyers, J.J., and Morimoto, R.I. (2002). The threshold for polyglutamine-expansion protein aggregation and cellular toxicity is dynamic and influenced by aging in Caenorhabditis elegans. Proc. Natl. Acad. Sci. U.S.A. *99*, 10417–10422. https://doi.org/10.1073/pnas.152161099.

12.	Aljohani, M.D., El Mouridi, S., Priyadarshini, M., Vargas-Velazquez, A.M., and Frøkjær-Jensen, C. (2020). Engineering rules that minimize germline silencing of transgenes in simple extrachromosomal arrays in C. elegans. Nat Commun *11*, 6300. https://doi.org/10.1038/s41467-020-19898-0.

13.	Dernburg, A.F., Zalevsky, J., Colaiácovo, M.P., and Villeneuve, A.M. (2000). Transgene-mediated cosuppression in the C. elegans germ line. Genes Dev *14*, 1578–1583.

14.	Parsian, A.J., Sheren, J.E., Tao, T.Y., Goswami, P.C., Malyapa, R., Van Rheeden, R., Watson, M.S., and Hunt, C.R. (2000). The human Hsp70B gene at the HSPA7 locus of chromosome 1 is transcribed but non-functional. Biochim Biophys Acta *1494*, 201–205. https://doi.org/10.1016/s0167-4781(00)00203-7.

15.	Urban, N.D., Lacy, S.M., Pelt, K.M.V., Abdon, B., Mattiola, Z., Klaiss, A., Tabler, S., and Truttmann, M.C. (2025). Functionally diversified BiP orthologs control body growth, reproduction, stress resistance, aging, and ER-Phagy in Caenorhabditis elegans. Preprint at bioRxiv, https://doi.org/10.1101/2025.01.14.633073 https://doi.org/10.1101/2025.01.14.633073.

16.	Ham, H., Woolery, A.R., Tracy, C., Stenesen, D., Krämer, H., and Orth, K. (2014). Unfolded protein response-regulated Drosophila Fic (dFic) protein reversibly AMPylates BiP chaperone during endoplasmic reticulum homeostasis. J. Biol. Chem. *289*, 36059–36069. https://doi.org/10.1074/jbc.M114.612515.

17.	Preissler, S., Rato, C., Chen, R., Antrobus, R., Ding, S., Fearnley, I.M., and Ron, D. (2015). AMPylation matches BiP activity to client protein load in the endoplasmic reticulum. Elife *4*, e12621. https://doi.org/10.7554/eLife.12621.

18.	Casey, A.K., Moehlman, A.T., Zhang, J., Servage, K.A., Krämer, H., and Orth, K. (2018). Fic-mediated deAMPylation is not dependent on homodimerization and rescues toxic AMPylation in flies. J Biol Chem *293*, 1550. https://doi.org/10.1074/jbc.AAC118.001798.

19.	Zinszner, H., Kuroda, M., Wang, X., Batchvarova, N., Lightfoot, R.T., Remotti, H., Stevens, J.L., and Ron, D. (1998). CHOP is implicated in programmed cell death in response to impaired function of the endoplasmic reticulum. Genes Dev *12*, 982–995.

20.	Sternberg, P.W., Van Auken, K., Wang, Q., Wright, A., Yook, K., Zarowiecki, M., Arnaboldi, V., Becerra, A., Brown, S., Cain, S., et al. (2024). WormBase 2024: status and transitioning to Alliance infrastructure. Genetics *227*, iyae050. https://doi.org/10.1093/genetics/iyae050.

21.	Zha, J., Ying, M., Alexander-Floyd, J., and Gidalevitz, T. (2019). HSP-4/BiP expression in secretory cells is regulated by a developmental program and not by the unfolded protein response. PLoS Biol *17*, e3000196. https://doi.org/10.1371/journal.pbio.3000196.

22.	The Alliance of Genome Resources Consortium (2020). Alliance of Genome Resources Portal: unified model organism research platform. Nucleic Acids Research *48*, D650–D658. https://doi.org/10.1093/nar/gkz813.

23.	Shen, X., Ellis, R.E., Lee, K., Liu, C.Y., Yang, K., Solomon, A., Yoshida, H., Morimoto, R., Kurnit, D.M., Mori, K., et al. (2001). Complementary signaling pathways regulate the unfolded protein response and are required for C. elegans development. Cell *107*, 893–903. https://doi.org/10.1016/s0092-8674(01)00612-2.

24.	Harding, H.P., Zhang, Y., Zeng, H., Novoa, I., Lu, P.D., Calfon, M., Sadri, N., Yun, C., Popko, B., Paules, R., et al. (2003). An Integrated Stress Response Regulates Amino Acid Metabolism and Resistance to Oxidative Stress. Molecular Cell *11*, 619–633. https://doi.org/10.1016/S1097-2765(03)00105-9.

25.	Safra, M., Ben-Hamo, S., Kenyon, C., and Henis-Korenblit, S. (2013). The ire-1 ER stress-response pathway is required for normal secretory-protein metabolism in C. elegans. J Cell Sci *126*, 4136–4146. https://doi.org/10.1242/jcs.123000.

26.	Tabara, H., Hill, R.J., Mello, C.C., Priess, J.R., and Kohara, Y. (1999). pos-1 encodes a cytoplasmic zinc-finger protein essential for germline specification in C. elegans. Development *126*, 1–11. https://doi.org/10.1242/dev.126.1.1.

27.	Farley, B.M., Pagano, J.M., and Ryder, S.P. (2008). RNA target specificity of the embryonic cell fate determinant POS-1. RNA *14*, 2685–2697. https://doi.org/10.1261/rna.1256708.

28.	Tabara, H., Sarkissian, M., Kelly, W.G., Fleenor, J., Grishok, A., Timmons, L., Fire, A., and Mello, C.C. (1999). The *rde-1* Gene, RNA Interference, and Transposon Silencing in *C. elegans*. Cell *99*, 123–132. https://doi.org/10.1016/S0092-8674(00)81644-X.

29.	Taylor, M.N., Spandana Boddu, S., and Vega, N.M. (2022). Using Single-Worm Data to Quantify Heterogeneity in Caenorhabditis elegans-Bacterial Interactions. J Vis Exp. https://doi.org/10.3791/64027.

30.	Brunquell, J., Bowers, P., and Westerheide, S.D. (2014). Fluorodeoxyuridine enhances the heat shock response and decreases polyglutamine aggregation in an HSF-1-dependent manner in Caenorhabditis elegans. Mech Ageing Dev *141–142*, 1–4. https://doi.org/10.1016/j.mad.2014.08.002.

31.	Feldman, N., Kosolapov, L., and Ben-Zvi, A. (2014). Fluorodeoxyuridine improves Caenorhabditis elegans proteostasis independent of reproduction onset. PLoS One *9*, e85964. https://doi.org/10.1371/journal.pone.0085964.
